# Impact of the R292K Mutation on Influenza A (H7N9) Virus Resistance towards Peramivir: A Molecular Dynamics Perspective

**DOI:** 10.3390/molecules27051645

**Published:** 2022-03-02

**Authors:** Sphamandla E. Mtambo, Samuel C. Ugbaja, Hezekiel M. Kumalo

**Affiliations:** Drug Research and Innovation Unit, Discipline of Medical Biochemistry, School of Laboratory Medicine and Medical Science, University of KwaZulu-Natal, Durban 4000, South Africa; sphamtambo@gmail.com (S.E.M.); ugbajasamchii@yahoo.com (S.C.U.)

**Keywords:** influenza A, H7N9, neuraminidase, peramivir, principal components analysis

## Abstract

In March 2013, a novel avian influenza A (H7N9) virus emerged in China. By March 2021, it had infected more than 1500 people, raising concerns regarding its epidemic potential. Similar to the highly pathogenic H5N1 virus, the H7N9 virus causes severe pneumonia and acute respiratory distress syndrome in most patients. Moreover, genetic analysis showed that this avian H7N9 virus carries human adaptation markers in the hemagglutinin and polymerase basic 2 (PB2) genes associated with cross-species transmissibility. Clinical studies showed that a single mutation, neuraminidase (NA) R292K (N2 numbering), induces resistance to peramivir in the highly pathogenic H7N9 influenza A viruses. Therefore, to evaluate the risk for human public health and understand the possible source of drug resistance, we assessed the impact of the NA-R292K mutation on avian H7N9 virus resistance towards peramivir using various molecular dynamics approaches. We observed that the single point mutation led to a distorted peramivir orientation in the enzyme active site which, in turn, perturbed the inhibitor’s binding. The R292K mutation induced a decrease in the interaction among neighboring amino acid residues when compared to its wild-type counterpart, as shown by the high degree of fluctuations in the radius of gyration. MM/GBSA calculations revealed that the mutation caused a decrease in the drug binding affinity by 17.28 kcal/mol when compared to the that for the wild-type enzyme. The mutation caused a distortion of hydrogen bond-mediated interactions with peramivir and increased the accessibility of water molecules around the K292 mutated residue.

## 1. Introduction

The seasonal upsurge of infections and pandemics from influenza viruses continues to cause death and morbidity. This also poses great economic stress and decreases productivity, especially in countries with temperate and tropical weather conditions [1,2]. The influenza virus is classified as *Orthomyxoviridae*, with classes A and B responsible for pandemic upsurges in humans [3,4]. The influenza virus contains three membrane proteins, i.e., matrix protein (M2), hemagglutinin (HA), and neuraminidase (NA) [5]. Hitherto, there are 9 NA and 16 HA of influenza A virus (IAV) subtypes, found especially in birds (aquatic), which have been responsible for major pandemic upsurges in humans [2]. Furthermore, there are other potentially pandemic IAV subtypes such as H5N1, H7N9, and H9N,2 which are transmitted to humans through aerosols [6]. IAV transmitted via aerosols are responsible for respiratory flu-like disorders especially among children, older persons, and persons with weakened immune system [7].

The emergence of the H7N9 subtype and its subsequent infection to humans were reported in March 2013 in China [7]. It was further reported within four months of its discovery, that about 130 persons were infected [8]. In January 2017, the World Health Organization recorded about 106 H7N9 human infections in China [8]. The World Health Organization had earlier postulated that there are about 250,000–500,000 global annual deaths due to influenza viruses; however, a recent study estimated higher figures of 290,000–650,000 global annual deaths from influenza, approximated to one billion people annually [9,10]. In 2019, it was reported that about 99,000–200,000 deaths resulted from respiratory tract influenza-related illnesses in populations less than 65 years old [10].

Recently, antiviral medications and vaccinations have been effectively employed in the fight against the influenza virus [11]. There are two major classes of anti-IAV drugs approved for the treatment of IAV diseases, i.e., neuraminidase (NA) inhibitors and adamantanes [1]. The ready availability of anti-IAV drugs gives them an edge over vaccines, which require a longer time to be developed [12]. Following the emergence of mutations and subsequent resistance of IAV to adamantane drugs, NA drugs such as laninamivir, zanamivir, oseltamivir, and peramivir have been widely employed for the treatment of IAV infections. Scientists are worried about the growing resistance of IAVs, nevertheless, NA appear to be the only type of IAV inhibitors currently in circulation [1]. Neuraminidases remain the preferred drugs for treating IAV, therefore resistance to the drug constitutes a setback in the fight against influenza pandemics [13]. The influenza A virus is known to produce diverse antigens through two processes. First, antigenic shifting due to the reassortment of the genetic compartments of two different IAVs in the same person, which results in a new strain of the virus; secondly, antigenic drifting in neuraminidase and hemagglutinin, which results in novel antigenic species. This is significantly facilitated by the imperfect nature of the viral polymerase [9]. Presently, the resistance to NA inhibitors is not very common, but might surface due to frequent use and administration of these drugs to the vulnerable groups [13]. Therefore, increased observation and additional studies on the mode of mutation/transmission are imperative.

**Figure 1:**
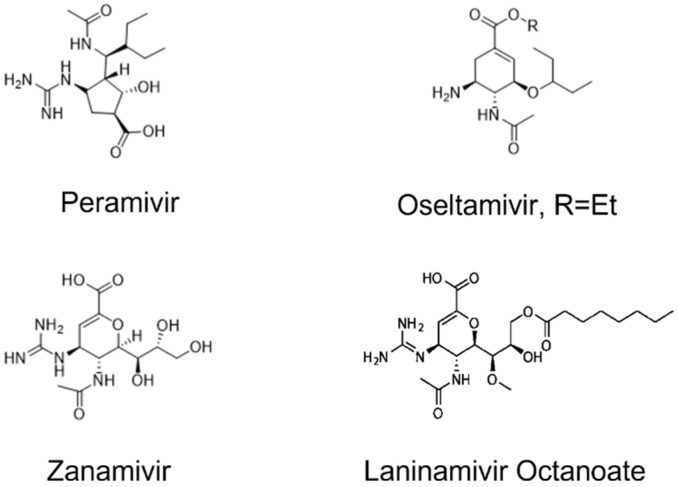
Structure of neuraminidase inhibitors.

The Food and Drug Administration lately approved an alternative intravenous NA drug called peramivir in addition to zanamivir and oseltamivir (Figure 1). Peramivir is a cyclopentane molecule that exhibited high selectivity and potency for influenza virus NA. In a recent study by Hseir et al. [14] the efficacy of intravenous peramivir over oseltamivir was investigated in the emergency department, and the results showed that influenza patients administered a single dose of peramivir showed a quick recovery [14]. In a laboratory study by Aoki et al., an in vitro influenza virus resistant to peramivir was also investigated [1]. It was observed that resistance to peramivir is due to changes in the gene for hemagglutinin, related to dual resistance to zanamivir and oseltamivir [1].

Neuraminidase inhibitors function by inactivating the viral NA enzyme [15]. NA is inactivated by blocking its ability to cleave sialic acid residues, thereby preventing the unleashing of the virus and stopping the infection of the host cells [15]. Neuraminidase inhibitors prevent the cleaving of sialic acid by the enzyme, and thereby virus spreading [16]. Several avian influenza subtypes (e.g., H5N1, H7N7, and H9N2) have caused human infections before. Their transmission has been controlled, possibly because avian viruses binding to the sialic acid receptors located in the human upper airways is inefficient [17]. NA inhibitors are sialic acid analogues that block the enzymatic active site and prevent its sialidase activity [18,19]. A single nucleotide change in the NA gene can generate resistance to NA inhibitors, as shown by the arginine-to-lysine amino acid mutation (R292K in N2 numbering, and R294K in N9 numbering) in the enzymatic active site. This R292K neuraminidase mutation has been reported in patients infected with H7N9 influenza A viruses and treated with NA inhibitors [20,21].

H7N9 normally circulates amongst avian virus populations with some variants known to occasionally infect humans [21,22]. Novel H7N9 viruses cause a severe respiratory disease in humans and have infected 1565 humans; about 39% of the people confirmed to be infected with Asian H7N9 virus have died since its emergence in 2013 [23]. Because of the lack of immunity against H7 subtype influenza viruses in the human population, the H7N9 virus is of concern as a potential cause of a pandemic [7]. The novel R292K variant virus has mammalian adaptation mutations in the receptor-binding site of the hemagglutinin gene and the polymerase basic 2 (PB2) gene (E627K) of the virus and can spread from poultry to man more easily [24].

Because of H7N9 resistance to the M2-ion channel blockers such as amantadine and rimantadine, neuraminidase inhibitors have been widely used for the antiviral treatment of patients with H7N9 [25,26]. Influenza virus H7N9 isolates (A/Anhui/1/2013, A/Shanghai/1/2013, and A/Shanghai/2/2013) were found to carry the NA-R292K mutation. This R292K mutation was determined to confer resistance to the inhibitory action of peramivir, which significantly impairs NA catalytic activity and virus replication in vitro and in vivo [26].

**Figure 2:**
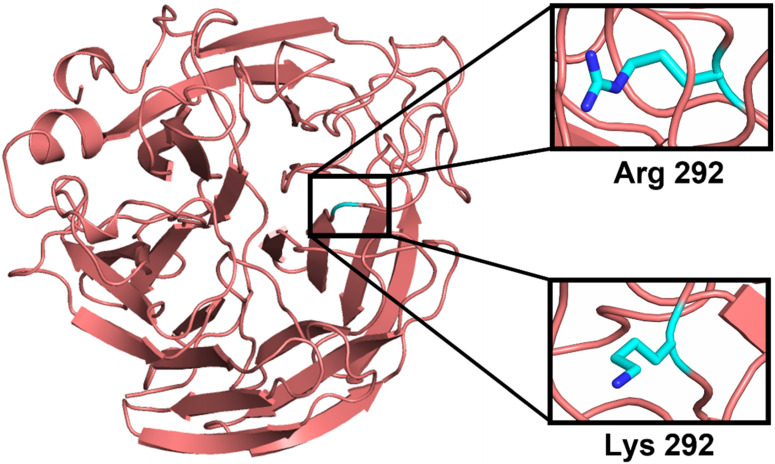
Representation of the R292K point mutation displaying the atomic Arginine and Lysine at residue number 292.

R292 is one of three key conserved arginine residues in the active site that surrounds the carboxylate group of sialic acid [27]. Neuraminidase inhibitors such as oseltamivir, peramivir, and zanamivir often interact with sialic acid at the enzyme’s active sites. This three-arginine cluster is a major factor for distorting the sialic acid pyranose ring from a chair to a boat conformation, a critical step for the hydrolytic cleavage of terminal sialic acid from adjacent membrane glycoproteins by influenza virus NA [4]. Peramivir contains a C4-guanidino group and a bulky hydrophobic pentyl ether side chain, like zanamivir and oseltamivir, respectively. These features lead to multiple interactions (higher binding affinity) with the NA catalytic site [28,29]. Features of the R292K mutant (Figure 2) and its contribution to peramivir resistance have not been deeply examined.

This present study was aimed at investigating the impact of the R292K mutation on H7N9 resistance towards peramivir at the interatomic level, as this promises to further elucidate other experimental studies previously conducted [27]. To achieve this, we employed the computational instruments of advanced molecular dynamic simulations [30]. We further investigated the intermolecular interactions between enzyme and ligand. The binding free energies of the peramivir-free neuraminidase and of the peramivir–wild-type neuraminidase and peramivir–R292K neuraminidase complexes were analyzed to unravel the molecular dynamics affecting the binding of peramivir. Understanding the molecular basis of resistance caused by such deleterious mutations is critical for the development of more effective anti-influenza virus compounds.

## 2. Results and Discussion

### 2.1. Root-Mean-Square Deviations (RMSD)

**Figure 3:**
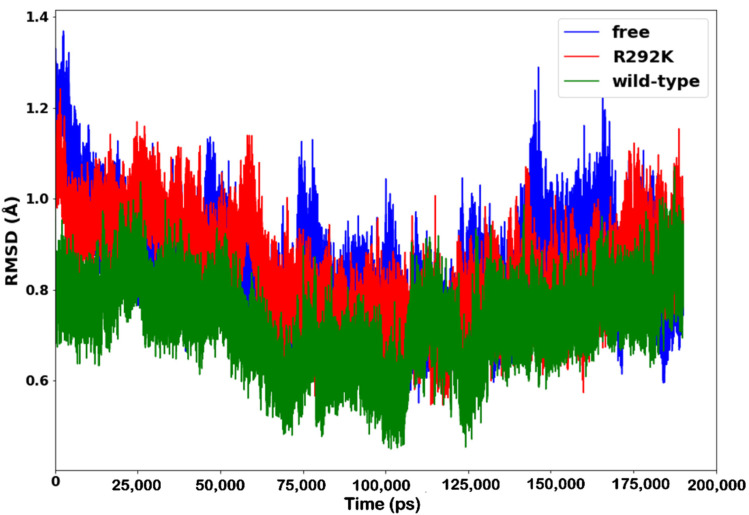
RMSD plot of C-α atoms of peramivir-free neuraminidase and peramivir–wild-type and peramivir–R292K neuraminidase complexes over simulation time (ps).

The RMSD of backbone C-α atoms was calculated to analyze the conformational stability of peramivir-free neuraminidase and peramivir-bound (wild-type and R292K) neuraminidase complexes and to observe the alignment of all the protein frames with that of the reference frame backbone. This type of analysis can yield information on the RMSD evolution of a protein and offer insights into its structural conformation throughout the simulation [31]. Figure 3 shows the RMSD values computed for peramivir-free neuraminidase and peramivir-bound wild-type and R292K neuraminidase complexes as a function of time. RMSD remained stable over most of the 200 ns simulation, thus providing a suitable basis for further analyses. The peramivir-free simulation showed relatively higher mean RMSD values, followed by peramivir–R292K variant complex and lastly by the peramivir-wild-type neuraminidase complex. A higher average RMSD for peramivir-free neuraminidase indicated higher flexibility of the molecule due to the absence of peramivir, resulting in an increased freedom for protein movement [31]. The higher average RMSD for the peramivir–R292K variant complex, when compared to that of the peramivir–wild-type neuraminidase complex demonstrated the flexibility of peramivir interaction with the protein and consequent interference with the active site structural framework, causing protein instability.

### 2.2. Root-Mean-Square Fluctuation (RMSF) and B Factors

**Figure 4:**
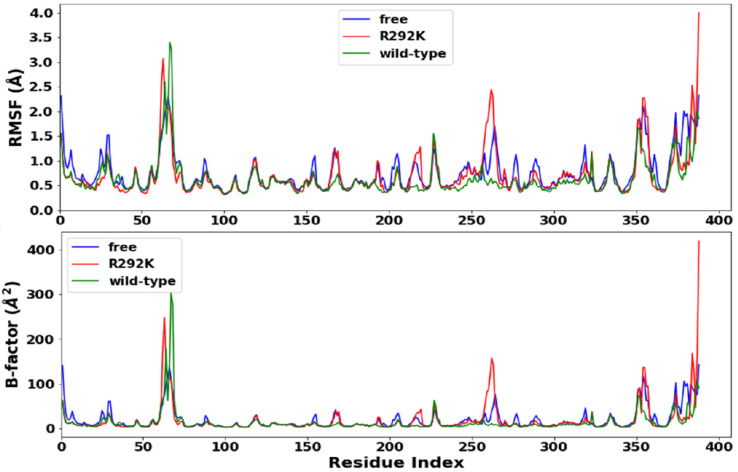
RMSF (**top**) and B-factors (**bottom**) plots of C-α atoms of peramivir-free neuraminidase, peramivir–wild-type, and peramivir–R292K neuraminidase complexes over simulation time (ps).

Figure 4 shows the per-residue C-α root-mean-square fluctuations (RMSF) and the atomic temperature factor (B-factor) of the simulations of peramivir-free neuraminidase and peramivir-bound (wild-type and R292K) neuraminidase complexes, calculated to gain an insight into the conformational flexibility of the overall residues in peramivir-free neuraminidase and peramivir-bound neuraminidase (wild-type and R292K) complexes. Fluctuations can indirectly lead to significant conformational changes in the active site and affect the dynamics of the protein, ultimately resulting in reduced functionality [32,33,34].

The average RMSF for all the amino acid residues was found to be 0.63 Å for the peramivir complex wild-type virus, 0.71 Å for the complex with the R292K variant, and 0.76 Å for the peramivir-free H7N9. A higher average RMSF for the peramivir-free virus indicated conformational instability because of the absence of peramivir (Figure 4). The R292K mutation was also found to impact the dynamics of some amino acid regions, i.e., 160–170, 190–200, 210–230, 260–270, and 350–370, when compared to the wild-type molecule, with the R292K variant having more fluctuations. Amino acid residues in the region 280–320 which contains the mutation site at position 292 showed higher fluctuation in the variant compared with the wild-type molecule. These fluctuations are suggested to be due to differences between the interactions of Arg or Lys side-chain atoms with surrounding molecules, and these differences may lead to a conformational disproportion between the wild-type and the R292K protein.

The atomic temperature factor (B-factor) measures the dynamic disorder caused by the temperature-dependent vibration of an atom, as well as the static disorder resulting from subtle structural differences in different unit cells throughout the crystal. It is very important to inspect the B-factors during a structural analysis. The identified flexible regions with a high average B-factor were found in peramivir-free neuraminidase (19.2 Å^2^), followed by the peramivir complex with the R292K variant (18.8 Å^2^) and lastly with wild-type neuraminidase (14.0 Å^2^). These results are in overall agreement with the RMSF-based flexibility analysis, suggesting that the absence of peramivir affects the overall conformational dynamics of the enzyme and that the R292K variant has significant conformational instability in the active site which results in reduced functionality.

### 2.3. Radius of Gyration (RoG)

**Figure 5:**
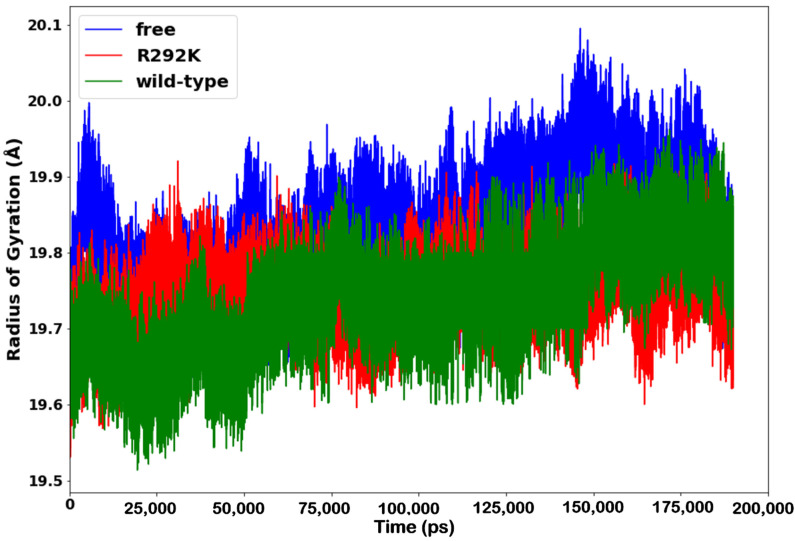
Radius of gyration plot of C-α atoms of the peramivir-free enzyme and peramivir–wild-type, and peramivir–R292K neuraminidase complexes over the simulation time (ps).

The radius of gyration yields quantitative measures such as folding, compactness, and shape of the protein in a biological system along with the MD simulation [35]. The assessment of protein collapse dynamics for the peramivir-free neuraminidase and the peramivir–(wild-type and R292K) neuraminidase complexes was performed as a function of time, as shown in Figure 5. The radius of gyration of the wild-type complex was significantly higher in comparison to that of the R292K complex and of peramivir-free neuraminidase throughout the simulation period. This indicates that the wild-type complex exhibits an overall more stable conformation than both the R292K complex and the peramivir-free enzyme. However, when comparing the stability of peramivir-free neuraminidase and the R292K complex, it was observed that the R292K complex was less compact than the peramivir-free enzyme. This indicates that the mutation of Arg292 to Lys292 decreased the compactness, negatively affecting the folding of the protein relative to the wild-type molecule. As the mutant structure became less compact, the interaction among neighboring amino acids decreased, which led to an unstable moment of inertia of the group of atoms from their center of mass. Such evidence implies that the mutant exhibits high conformational flexibility which decreases the receptor–ligand stability. The fluctuation of the radius of gyration is in agreement with the RMSF and the B-factor determined in the flexibility studies.

### 2.4. MM/GBSA Binding Free Energy Calculation

All molecular mechanics and solvation energy components were calculated using the MM/GBSA approach over a 200 ns MD trajectory, as listed in Table 1.

The calculated binding free energy (ΔG_bind_) for the wild-type neuraminidase complex was −38.95 ± 5.66 kcal/mol, while that of the R292K mutant was −21.67 ± 2.10 kcal/mol. Such a large reduction in binding affinity (−17.28 kcal/mol) due to thermodynamic instability of the protein–ligand complex could impair drug binding and thus reduce the effectiveness of peramivir against the mutant. These calculations agree with experimental data that indicated that the R292K mutation leads to a 563-fold increased relative resistance towards peramivir [26]. Peramivir comprises a C4-guanidino group and a bulky hydrophobic pentyl ether side chain. These features lead to higher binding affinity with the NA binding site [27]. Thus, the R292K mutation in the binding site might reduce peramivir binding affinity.

The calculated van der Waals contributions (ΔE_vdW_) to the binding free energy in the wild-type neuraminidase complex (−38.28 ± 2.33 kcal/mol) were lower than those in the R292K mutant neuraminidase complex (−28.43 ± 3.49 kcal/mol). On the other hand, the calculated electrostatic contributions (Δ*E*_ele_) to the binding free energy for the R292K mutant neuraminidase complex (−81.29 ± 8.85 kcal/mol) were higher compared to those for the wild-type neuraminidase complex (−104.55 ± 15.85 kcal/mol). The free energy components presented in Table 1 suggest that Δ*E*_vdW_ and Δ*E*_ele_ are the major energy contributors to peramivir binding. This is due to the amino acid residues present in the binding site of the wild-type molecule, exhibiting strong hydrophobic interactions and thus stabilizing the conformation of the protein–ligand complex.

The calculated solvation energy (Δ*G*_sol_) of the wild-type neuraminidase complex (105.12 ± 14.5 kcal/mol) was higher than that of the R292K mutant neuraminidase complex (92.57 ± 7.22 kcal/mol). The significant difference in the Δ*G*_sol_ (12.55 kcal/mol) resulting in weak intermolecular interactions between the neuraminidase complex and water molecules also confirmed that the R292K mutation has the potential to significantly affect the efficacy of peramivir against H7N9 avian influenza virus. Due to limitations associated with approximations in the binding free energy calculations, the binding free energy values represent a trend for the wild-type and the mutant binding free energies.

### 2.5. Hydrogen Bond Formation

One of the most important analyses is that of the number of hydrogen bonds between residues to evaluate the stability of a protein. A high number of intermolecular hydrogen bonds in a protein might help to maintain its rigidity, while a low number of hydrogen bonds with a solvent makes the protein more flexible. The introduction of a single mutation in a protein is expected to cause changes in hydrogen bonds around the site of mutation. Therefore, we examined hydrogen bond formation during the simulation for peramivir-free neuraminidase and the peramivir–(wild-type and R292K) neuraminidase complexes.

To further examine the effect of the R292K mutation on peramivir binding, we monitored hydrogen bond distances (Å) and hydrogen bond occupancy (%) between amino acid residues interacting with peramivir in the active site of the wild-type and R292K mutant neuraminidase complexes. A summary of the average hydrogen bond distances and occupancy attained during simulation is presented in Table 2. Hydrogen bonds were recorded throughout the 200 ns trajectory. Hydrogen bonds of the mutant complex exhibited an increase in average distance when compared to the wild-type complex. The wild-type complex showed a higher occupancy of hydrogen bonds when compared to the mutant complex throughout the simulation. This indicated a strong attraction interaction between active site residue atoms and peramivir. It also showed that the mutation affects the hydrogen bond network of the complex. The long-distance hydrogen bond network causes a loss of hydrogen bond interactions between the active site residues and peramivir, which in turn leads to structural instability and eventually affects peramivir binding.

**Figure 6:**
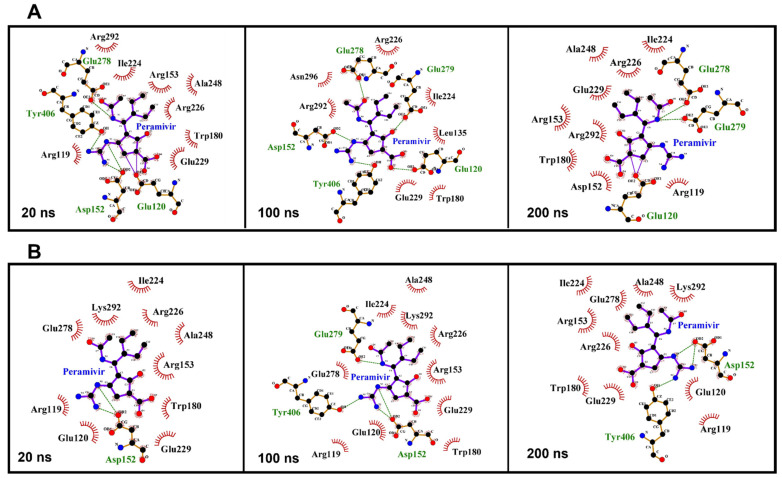
Amino acid residue interactions with peramivir in the active site of the wild-type (**A**) and R292K mutant (**B**) at 20 ns, 100 ns, and 200 ns simulation time.

LigPlot software was used to analyze the interaction between peramivir bound to wild-type and R292K mutant neuraminidase (Figure 6) [36]. Certain amino acid residues elicited strong hydrogen interactions with peramivir, which accounted for the enzyme stability and high affinity towards peramivir. As illustrated in Figure 6A, among all interactions between peramivir and wild-type NA, certain amino acid residues consistently established interactions with peramivir throughout the simulation. Notable amongst these residues are Glu 120 and Glu 278, which consistently maintained a hydrogen bond interaction with peramivir at 10 ns, 100 ns, and 200 ns. These hydrogen bond interactions, in addition to the many hydrophobic interactions with other active site residues, could jointly contribute to the favorable binding free energies that we calculated. The binding of peramivir to R292K mutant NA was also characterized by consistent hydrogen bond interactions with some specific residues throughout the simulation, but Glu 120 and Glu 278 were not involved in these hydrogen bond interactions (Figure 6B). This suggests that the R292K mutation induced a loss of hydrogen bond interactions between peramivir and Glu 120 as well as Glu 278, thus resulting in a reduction of the binding affinity of peramivir to the active site. This could imply that interactions of these amino acids with peramivir could be significant for the high-affinity binding and stability of the peramivir–NA complex.

**Figure 7:**
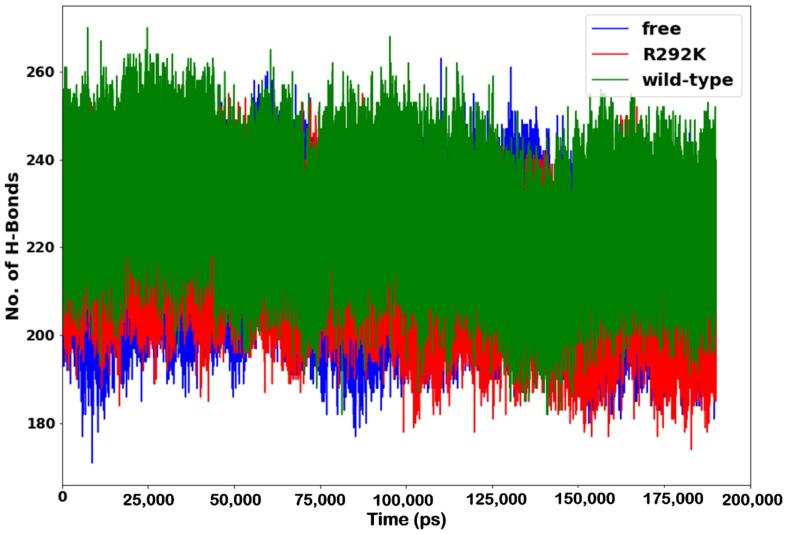
Number of H-bond formation in peramivir-free neuraminidase and the peramivir–wild-type and peramivir–R292K neuraminidase complexes over the simulation time (ps).

The analysis of hydrogen bond formation between amino acid residues indicated that the wild-type complex displayed a relatively higher hydrogen bond participation with other amino acids and comparatively less flexibility over the simulation time when compared to the R292K complex and peramivir-free neuraminidase (Figure 7). Based on the observed RMSF values and number of hydrogen bonds, it was confirmed that the mutation led to a more flexible conformation due to the formation of a smaller number of hydrogen bonds. The lower number of hydrogen bond interactions between amino acid residues in the R292K variant led to a distinct reduction in the peramivir binding affinity due to conformational distortion and, as such, to a decrease in receptor–ligand interaction.

### 2.6. Solvent-Accessible Surface Area (SASA)

**Figure 8:**
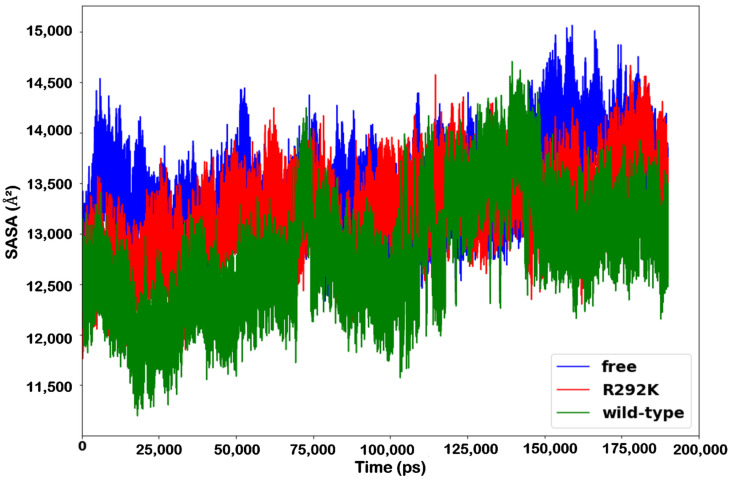
SASA (Å^2^) plot of peramivir-free neuraminidase and the peramivir-wild-type and peramivir- R292K neuraminidase complexes over the simulation time (ps).

The interactions of a protein with various solvents and ligands depend primarily on its surface properties. Thus, understanding the changes in solvent-accessible surface area (SASA) due to structural deviations can be important. SASA is the total amount of surface area available for interacting with other ligands, proteins, or solvents and is used to characterize the compactness of protein structures. The plot of solvent-accessible surface area (SASA) of peramivir-free neuraminidase and peramivir-bound (wild-type and R292K) neuraminidase complexes against time at 300 K is shown in Figure 8. Major fluctuations were observed throughout the simulation time. It was evident that SASA for the peramivir-free neuraminidase was higher compared to those of the R292K variant and the wild-type complexes. Also, when comparing SASA between the wild-type and R292K variant complexes, the latter had higher SASA. The higher values of SASA for the peramivir–R292K complex indicated that the variant structure is thermodynamically unstable. This is due to the Arg292 mutation causing the protein structure to become less compact and thus exposing more protein to water molecules. Therefore, the SASA induced by the R292K mutation markedly influences the structure as well as the activity of neuraminidase.

### 2.7. Principal Components Analysis (PCA)

**Figure 9:**
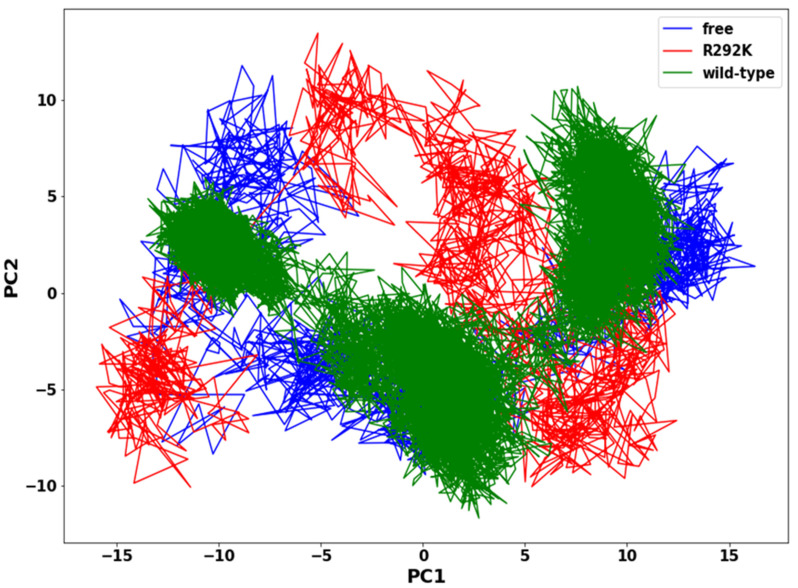
PCA scatter plot projection of C-α atoms motion of the first two principal components, PC1 and PC2, for peramivir-free neuraminidase and peramivir–wild-type and peramivir–R292K neuraminidase complexes conformations.

The assessment of the functionally relevant global aggregate motion of a protein is a very demanding task. However, PCA helps in reducing the complexity of classifying collective motions of a protein, since it segregates global aggregate motions from local fast motions. These essential movements in a protein are directly interconnected to protein stability and therefore associated with protein function. The 2D projection of the trajectory plot elucidates the overall collective motion of a protein in the essential subspace of the system. The flexibility of peramivir-free neuraminidase, peramivir-bound wild-type, and peramivir-bound R292K neuraminidase complexes was assessed using the PCA method, showing a significant difference between the systems and indicating a difference in protein motion (Figure 9). In the PCA plot, we observed that the wild-type complex occupies a lower subspace in comparison to peramivir-free neuraminidase and the R292K complex. The highly compact and stable structure of the wild-type complex makes the residue side-chain atoms fluctuate in a smaller subspace when compared to the variant complex and peramivir-free neuraminidase. Such evidence suggests that the mutant structure has a high degree of flexibility which disturbs the binding interaction with peramivir.

## 3. Materials and Methods

### 3.1. System Preperation

The X-ray crystal structure of the Anhui N9–peramivir (PDB code: 4MWV) complex was retrieved from the Protein Data Bank (http://www.rcsb.org, accessed on 6 September 2021). The crystal structure of neuraminidase established by X-ray showed that it exists as a homo-tetramer. However, only one chain (chain A) was used for simulations in this study to reduce the computational cost. The Arginine to Lysine point mutation 292 (R292K) was introduced using PyMol (Version 2.5) [37]. Ligand and receptor were modified and visualized by PyMol and Avogadro software (Version 1.2) [38], respectively.

### 3.2. Molecular Dynamic Simulations

Molecular dynamic simulations of peramivir-free neuraminidase and peramivir–neuraminidase complexes (wild-type and R292K) were performed using Amber 14 software package [39,40,41]. Gaussian 09 at the HF/6–31G* level was utilized to optimize the geometry for the ligand. The antechamber module was used for the generation of atomic partial charges for the ligand using Restrained Electrostatic Potential (RESP) and the General Amber Force Field (GAFF) procedures [42]. The ff99SB force field in the Amber 14 suite was used to define the parameters of the protein system [43]. Missing hydrogen and heavy atoms were added using the LEAP module of AMBER 14. The system was neutralized by the addition of sodium ions. The entire system was solvated within a box of TIP3P [44] water molecules such that any solute atoms were within 10 Å of any box edge during the MD simulations. The periodic boundary conditions were adopted, and the long-range electrostatic interactions were treated with the Particle mesh Ewald (PME) method [45] with a direct space and van der Waals cut-off of 12 Å. Initial energy minimization of 2000 steps was carried out with a restraint potential of 500 kcal/mol Å applied to the solute, for 1000 steps using the steepest descent method followed by conjugate gradient minimization of 1000 steps. An additional full minimization of 1000 steps was carried out by unrestrained conjugate gradient. Gradual heating of the systems from 0 to 300 K with a 5 kcal/mol Å harmonic restraint potential and a Langevin thermostat of collision frequency of 1/ps using a canonical ensemble (NVT) molecular dynamics simulation were then carried out [46]. The systems were equilibrated at 300 K in an NPT ensemble for 500 ps without restraint. A Berendsen barostat was used to maintain the pressure of the systems at 1 bar. All hydrogen bonds were constrained using the SHAKE algorithm [47], and a time scale of 2 fs for all MD runs using the SPFP precision model [48] was applied. A 200 ns production run was performed without any restrain on the systems in an NPT ensemble at a temperature of 300 K with a target coupling constant of 2 ps and pressure at 1 bar. The coordinates were saved every 1ps time interval, and the trajectories were analyzed every 1 ps. The PTRAJ and CPPTRAJ modules [49] of Amber14 suite were utilized for post-dynamic analyses, such as root-mean-square deviation (RMSD) and root-mean-square fluctuations (RMSF), radius of gyration (RoG), solvent-accessible surface area (SASA), B-factor, hydrogen bond occupancy over time, and principal component analysis (PCA). All molecular visualization and plots were carried out using the PyMol system and Matplotlib data analysis tools, respectively [50].

### 3.3. Thermodynamic Calculations

The binding free energy profiles of the peramivir-bound neuraminidase (wild-type and R292K) complexes were computed using the Molecular Mechanics/Generalized Born Surface Area (MM/GBSA) approach [51,52,53,54]. The free energies of binding were calculated considering 1000 snapshots from the 200 ns trajectory. Binding free energy calculation is an endpoint energy calculation that offers a valuable insight into the formation of the protein–ligand complex. The following set of equations procide a detailed explanation of the calculation of binding free energy:

ΔG_bind_ = G_complex_ − G_receptor_ − G_ligand_ (1)

ΔG_bind_ = E_gas_ + G_sol_ − TΔS (2)

E_gas_ = E_int_ + E_vdW_ + E_ele_ (3)

G_sol_ = G_GB_ + G_SA_ (4)

G_SA_ = γSASA (5)

where G_complex_ is the total free energy of the protein–ligand complex, G_receptor_ and ∆G_ligand_ are total free energies of the isolated protein and ligand in the solvent, respectively [54]. E_gas_ signifies the gas-phase energy and is evaluated directly from the Amber ff99SB force field terms. G_sol_ denotes the solvation-free energy that can be decomposed into polar and nonpolar contribution states. T∆S refers to the entropic contribution to the free energy in a vacuum where T and S denote the temperature and entropy, respectively. E_int_ signifies the internal energy, E_ele_ is the intramolecular electrostatic energy, and E_vdW_ is the van der Waals energy. The solvation-free energy G_sol_ is the energy required to transfer a solute from the vacuum into a solvent. G_GB_ and G_SA_ are the electrostatic and non-electrostatic contributions to the solvation free energy, respectively. G_GB_ was computed using the Poisson–Boltzmann (PB) equation, and G_SA_ was estimated from the solvent-accessible surface area (SASA) equation estimated by using a water probe radius of 1.4 Å. T and S are the temperature and the total solute entropy, respectively; γ is a coefficient related to the surface tension of the solvent [55,56]. A constant γ = 0.0072 kcal/mol/Å2 was used with Amber PB polar solvation energies. The external and internal dielectric constants were set at 80 and 1, respectively.

### 3.4. Principal Components Analysis (PCA)

Before processing the MD trajectories for PCA, the 200 ns MD trajectories of peramivir-free neuraminidase and peramivir-bound neuraminidase (wild-type and R292K) complexes were stripped of solvent and ions using the PTRAJ module [50] of AMBER 14. The covariance matrix (C-α atoms) between residues i and j were calculated for each of the 200 ns MD simulation trajectories. PCA was performed on C-α atoms over 10 snapshots taken from trajectories at a time interval of 20 ps, overall translation and rotation trajectories were removed, and only C-α was kept for the analysis. To obtain collective motion coordinates that represented the overall dynamics of each trajectory, PCA was performed, in which the covariance matrix was diagonalized to yield a set of eigenvectors and eigenvalues. Using in-house scripts, the first two principal components (PC1 and PC2) were calculated, and covariance matrices were generated. The first two principal components correspond to the first two Eigenvectors of the covariance matrix. PCA scatter plots were then constructed using Matplotlib [57,58].

## 4. Conclusions

In this study, the impact of the single point mutation R292K on pathogenic H7N9 in influenza neuraminidase on peramivir binding to the enzyme was investigated using various computational approaches. These approaches, including MD simulations, principal component analysis, root-mean-square deviation, radius of gyration, and solvent-accessible surface area, aided us to understand the impact of the R292K mutation on resistance to peramivir. Our findings showed that the R292K mutation in H7N9 neuraminidase decreased the binding with peramivir, as shown by the high flexibility of peramivir RMSD and RMSF in the mutant pocket; a large radius of gyration of the mutant complex decreased the interaction among neighboring amino acid residues such that it reduced receptor–ligand clutching and increased the accessibility for water molecules around the K292 mutated residue and the carboxylate group of peramivir, thus disturbing the drug binding process.

The results of this work suggest that the bulky hydrophobic pentyl ether side chain of peramivir weakly binds to K292; thus, zanamivir and laninamivir with their hydrophilic bulky groups promise to be potent neuraminidase inhibitors. This study verified that the R292K mutation decreases peramivir binding affinity by 17.28 kcal/mol, distorts the ligand optimum orientation in the neuraminidase active site, affects the overall peramivir conformational shape, and distorts the hydrogen bond interaction network between enzyme and ligand. The findings of this study can be useful for the investigation of other mutations that can affect the effect of peramivir on H7N9 influenza A virus.

## Figures and Tables

**Table 1:** MM/GBSA binding free energies profile of peramivir bound to H7N9 wild-type neuraminidase and R292K mutant neuraminidase.

Complexes	ΔG_bind_	ΔE_ele_	ΔE_vdw_	ΔE_gas_	ΔG_sol_
Wild-type	−38.95 ± 5.66	−104.55 ± 15.85	−38.28 ± 2.33	−130.93 ± 13.66	105.12 ± 14.5
R292K	−21.67 ± 2.10	−81.29 ± 8.85	−28.43 ± 3.49	−112.02 ± 10.9	92.57 ± 7.22

ΔG_bind_—binding free energy; Δ*E*_ele_—electrostatic interaction; Δ*E*_vdW_—van der Waals forces; Δ*G*_gas_—gas–phase interaction; Δ*G*_sol_—solvation energy.

**Table 2:** Average hydrogen bond distances and percentage occupancy between amino acid residues interacting with peramivir calculated over the simulation time.

H-bond	Average Distance (Å)		Percentage Occupancy (%)	
	Wild-Type	Mutant	Wild-Type	Mutant
Glu120 (OE_2_)…(O_3_) Peramivir	2.84	-	7.1	-
Asp152 (OD_2_)…(N_3_) Peramivir	2.68	2.83	92.3	87.3
Asp152 (OD_2_)…(N_4_) Peramivir	2.89	2.93	72.4	67.2
Glu278 (OE_1_)…(O_1_) Peramivir	2.90	-	12.6	-
Glu279 (OE_2_)…(O_2_) Peramivir	2.79	2.94	68.7	60.8
Glu279 (OE_2_)…(N_1_) Peramivir	2.64	2.84	75.0	67.7
Tyr406 (OH)…(N_3_) Peramivir	2.81	2.95	89.5	80.2

## Data Availability

The data presented in this study are available on request from the corresponding author.

## Sample Availability

Not available.
